# Controlled growth of titanium dioxide nanotubes for doxorubicin loading and studies of *in vitro* antitumor activity

**DOI:** 10.3389/fbioe.2023.1201320

**Published:** 2023-05-11

**Authors:** Yunshan Zhang, Tuo Huang, Wanwan Lv, Kai Yang, Cuiling Ouyang, Minxin Deng, Rongyuan Yi, Hui Chu, Jian Chen

**Affiliations:** ^1^ Research Center for Intelligent Sensing Systems, Zhejiang Lab, Hangzhou, China; ^2^ School of Chemistry and Chemical Engineering, Hunan University of Science and Technology, Xiangtan, Hunan, China; ^3^ Fourth Department of Gynecologic Oncology, Hunan Cancer Hospital/The Affiliated Cancer Hospital of Xiangya School of Medicine, Central South University, Changsha, Hunan, China

**Keywords:** titanium dioxide nanotubes, anodization voltages, drug loading and release, cellular uptake, antitumor efficiency

## Abstract

Titanium dioxide (TiO_2_) materials are suitable for use as drug carriers due to their natural biocompatibility and nontoxicity. The aim of the study presented in this paper was to investigate the controlled growth of TiO_2_ nanotubes (TiO_2_ NTs) of different sizes via an anodization method, in order to delineate whether the size of NTs governs their drug loading and release profile as well as their antitumor efficiency. TiO_2_ NTs were tailored to sizes ranging from 25 nm to 200 nm according to the anodization voltage employed. The TiO_2_ NTs obtained by this process were characterized using scanning electron microscopy, transmission electron microscopy, and dynamic light scattering The larger TiO_2_ NTs exhibited greatly improved doxorubicin (DOX)-loading capacity (up to 37.5 wt%), which contributed to their outstanding cell-killing ability, as evidenced by their lower half-maximal inhibitory concentration (IC50). Comparisons were carried out of cellular uptake and intracellular release rates of DOX for large and small TiO_2_ NTs loaded with DOX. The results showed that the larger TiO_2_ NTs represent a promising therapeutic carrier for drug loading and controlled release, which could improve cancer treatment outcomes. Therefore, TiO_2_ NTs of larger size are useful substances with drug-loading potency that may be used in a wide range of medical applications.

## 1 Introduction

In various biomedical applications, biomaterials are anticipated to directly fulfill the need for materials in diagnosis, treatment, and bio-implants (Zhang et al., 2022). Research in the field of biomaterials has focused on nanotechnology and nanomaterials. Natural and biocompatible nanotubes (NTs) are among the best materials for delivery of drug nanoformulations (Dias-Netipanyj et al., 2019; Chen et al., 2021). In addition, the straightforward synthesis process and their large surface-to-volume ratios mean that they overcome many limitations of existing conventional materials (Kovrigina et al., 2022). For example, halloysite NTs are among the most versatile nanomaterials utilized in biomedical applications (Hu et al., 2017). These are commercially available layered aluminosilicates with hollow tubular geometry, synthesized in an appropriate diameter range (ca. 40–70 nm). Due to high lumens and abundant hydroxyl density on their surface, halloysite NTs have become a promising material for surface modification and drug loading. To obtain halloysite NTs of suitable size, ultrasonication is commonly used to cut long halloysite NTs to form short NTs. Synthesis of NTs made from polypyrrole, a series of compounds isolated from bone oil, is easily achieved by electrospinning, self-assembly, coprecipitation, and template methods. Polypyrrole NTs are also promising and versatile candidates as drug carriers due to their chemical grafting and stealth transport properties (Chen et al., 2017). In particular, polypyrrole NTs of an appropriate size can be acquired via a template synthesis route. Silica-based nanomaterials have also garnered significant attention because of their nontoxic nature (Huang et al., 2020). Silica NTs exhibit plasticity due to abundant Si–OH bonds on the surface and an empty inner space which can be filled with cargo. Desirable silica NTs of different thicknesses and dimensions have been fabricated by changing the reaction parameters, which will be beneficial for applications in biology and medicine. Carbon nanotubes, a one-dimensional material created by rolling up a sheet of graphene, are ideal carriers for the selective delivery of anticancer drugs in virtue of their transporting capabilities, which can be combined with appropriate surface modifications. However, there are some limitations to consider, such as low biocompatibility and water solubility (Maleki et al., 2020; Pistone et al., 2016). Thus, these tubular materials of suitable size may be loaded with drugs at a higher capacity (Wu et al., 2021). Narrow tube openings also allow for controllable and sustained drug release over the course of hours, days, or even weeks (Lvov et al., 2016). TiO_2_ NTs are a series of biocompatible tubular nanocarriers that exhibit well-separated and uniform NT morphology and superior mechanical properties (Cheranmadevi et al., 2023). Ideally, the size of the aforementioned NTs may be controlled by ultrasonication, by the template route, or by varying the synthesis parameters. Given that features such as size and surface morphology significantly affect the loading and release performance of drug-loaded NTs, their synthesis with desired experimental parameters has remained a topic of significant interest (Shidfar et al., 2017; Hasanzadeh Kafshgari et al., 2019). In addition, the side effects of antitumor drugs are a major issue in traditional chemotherapy, and these hollow tubular materials are an ideal way to reduce side effects and deliver the drugs for release at the tumor site (Lv et al., 2023). More importantly, because pH in the tumor matrix is usually more acidic than that of normal tissues and blood (with the lysosomes in tumor cells reaching an acidic of pH ∼ 4.5–5.0; Deng et al., 2022), TiO_2_ NTs with superior pH-sensitive features may be feasible for controlled drug release (Feng et al., 2016; Yi et al., 2022).

In particular, electrochemical anodization has been applied to prepare self-organized TiO_2_ nanotubular arrays on Ti substrates with controllable NT size (Tenkyong et al., 2018). In addition, such NT arrays are easily peeled and can be further functionalized by a variety of physical and chemical processes. Dispersive TiO_2_ NTs can be obtained via sonication of anodic NT arrays. This method offers a distinctive advantage since the morphology of the TiO_2_ NTs formed in situ can easily be tuned by adjusting the anodization parameters. Previous studies have demonstrated NT formation as follows (Fu and Mo, 2018; Zhao et al., 2018): first, the oxidation layer is formed by anodizing titanium films in an electrolyte. Second, the pores are formed by the chemical dissolution of TiO_2_ in the presence of fluoride ions. Third, the TiO_2_ NTs can easily be tuned to a very wide range of lengths and sizes by adjusting the anodization parameters, especially the anodization voltage. For example, Lockman et al. elucidated the effect of voltage on the dimensions of TiO_2_ NTs in an excess F^−^ bath and conducted anodization experiments at different voltages (Lockman et al., 2010). Francisco et al. reported that the desired dimensional modulation of TiO_2_ NTs can be performed, covering a broad range of voltages (Cortes et al., 2016). For this purpose, a successful strategy enables well-separated single NTs with different sizes based on the anodization voltage.

**SCHEME 1:**
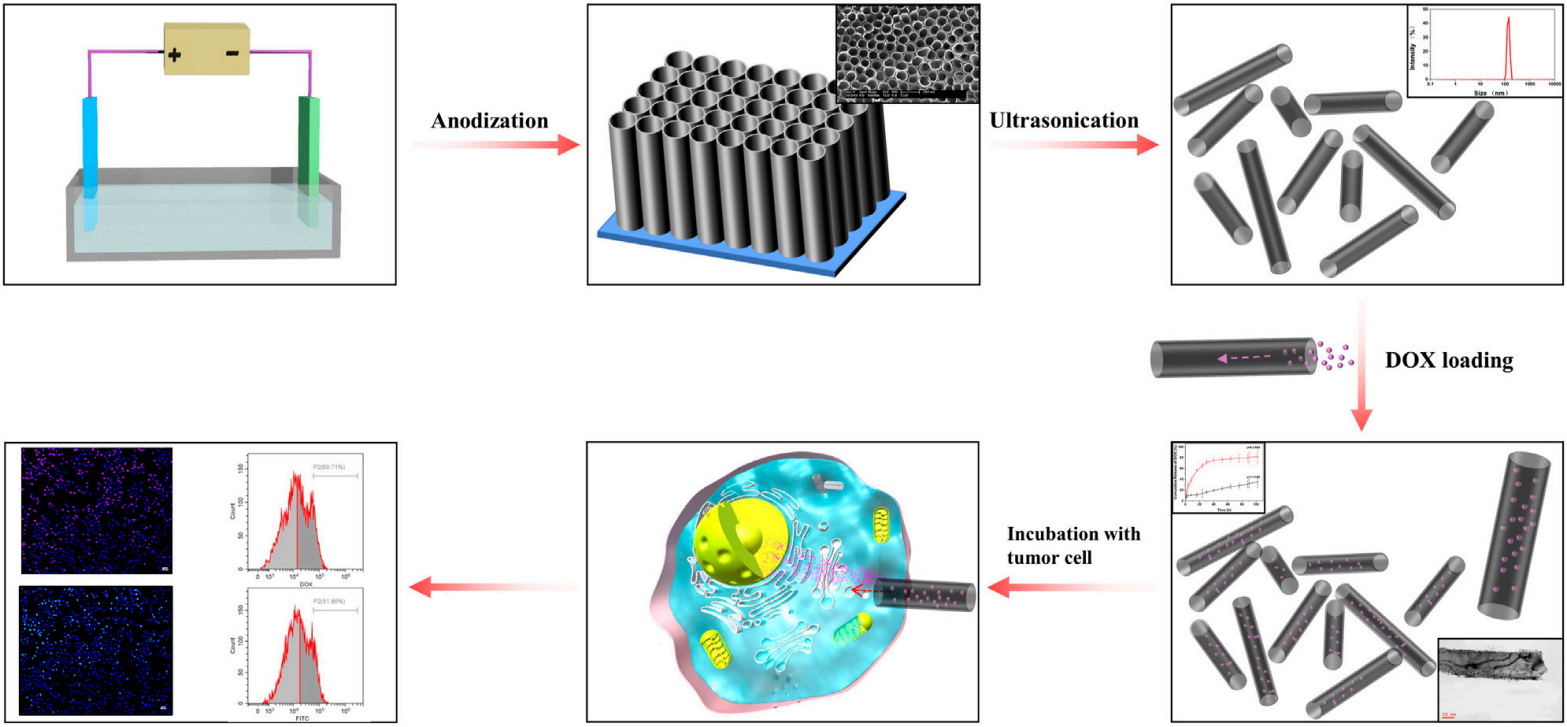
Representation of the preparation of size-controlled TiO_2_ NT nanocarriers and their application as a scaffold for drug loading and release.

It has been proposed that NT size plays a critical role in drug loading and release, as well as antitumor efficiency (Gulati et al., 2015). Therefore, in the present study, we focused on size-dependent regulation, selecting TiO_2_ NTs of different sizes for comparison of their drug loading and release properties, as well as their antitumor efficiency. Here, we also present a novel and straightforward method for synthesis of TiO_2_ NTs (Scheme 1). Through the anodization process, the sizes of the TiO_2_ NTs were governed by a voltage-dependent control. Subsequently, TiO_2_ NTs of different sizes were subjected to annealing and DOX loading. Dispersive TiO_2_ NTs were obtained from a Ti substrate via ultrasonication. The morphology and size of the as-prepared TiO_2_ NTs were investigated by SEM, TEM, and DLS. The potential performance of TiO_2_ NTs of different sizes in terms of drug loading and release ability was tested, and the cytotoxicity of DOX-loaded TiO_2_ NTs of different sizes was evaluated. Finally, intracellular uptake behaviors of DOX-loaded TiO_2_ NTs of different sizes were assessed.

## 2 Materials and methods

### 2.1 General

All reagents were purchased from commercial sources and used without further purification, unless otherwise stated. A scanning electron microscope (SEM; FEI Sirion 200) was used to observe the morphology of the support at 20 kV. Transmission electron microscopy (TEM) analyses were carried out using a JEOL-2100F instrument operating at 200 kV. Hydrodynamic size was measured by dynamic light scattering (DLS; Malvern ZEN 3600 Instrument).

### 2.2 Preparation of TiO_2_ NTs of different sizes

A mixed solution of 0.5 wt% NH_4_F, 10 vol% H_2_O, and 0.9 vol% H_2_SO_4_ in ethylene glycol was used as the electrolyte. Spotless Ti foils were immersed in the electrolyte for pre-treatment at room temperature for 1 h. Subsequently, the Ti foils were anodized at 50 V, 70 V, 100 V, 120 V, and 150 V for 10 min in each case. After anodization, the Ti foils were washed three times with H_2_O and dried at 60°C for 12 h. Subsequently, the samples were annealed at 450°C in air for 3 h to crystallization.

### 2.3 Preparation of DOX-loaded TiO_2_ NTs

The samples were scraped from the Ti substrates, then dispersed in PBS and ultrasonicated for 10 min to obtain monodisperse NTs. The resulting TiO_2_ NTs were dispersed in 25 mM DOX solution with stirring for 24 h at 30°C. After loading, the samples were centrifuged and rinsed with ddH_2_O to remove unloaded DOX. The suspensions were filtered using a microfiltration membrane (3 μm) to remove macroparticles and then centrifuged at 15,000 rpm for 15 min. The precipitates were taken out for drying.

### 2.4 DOX-loading capacity of TiO_2_ NTs and release from TiO_2_ NTs *in vitro*

DOX-loaded TiO_2_ NTs were placed in a dialysis bag, immersed in six-well plates with 1 ml release medium, and agitated at 50 rpm at 37°C. Subsequently, 1 ml of the release medium was removed for detection of the amount of DOX released using an ultraviolet-visible spectrophotometer (Shimadzu UV 2450, Japan) at 490 nm. After detection, 1 ml of the release medium was replaced in six-well plates for agitation. The measurements were repeated until no further increase in DOX was detected. The DOX-loading capacity was calculated according to a standard calibration curve. The linear equation was y = 0.01537x − 0.0127 (*R* ^2^ = 0.9985). For investigation of release properties, DOX-loaded TiO_2_ NTs (DOX-equivalent dose of 1 mg) were again placed in a dialysis bag; they were immersed in six-well plates with 2 ml release medium (pH = 6.5 and 7.4 PBS) and agitated at 50 rpm at 37°C. Next, 1 ml of release medium was extracted at predetermined time intervals (1 h, 2 h, 4 h, 8 h, and 102 h) for evaluation of the amount of DOX released, with a detection wavelength at 490 nm.

### 2.5 Evaluation of the cytocompatibility of TiO_2_ NTs of different sizes and the cytotoxicity of DOX-loaded TiO_2_ NTs of different sizes

SKOV-3 cells were grown in RPMI 1640 medium (Thermo Fisher Scientific, Waltham, MA, United States) supplemented with 10% fetal bovine serum (FBS) (Gemini Bio Products, West Sacramento, CA, United States). The cytocompatibility of the prepared TiO_2_ NTs was assessed by CCK-8 assay. For this purpose, 1 × 10^4^ SKOV-3 cells were seeded on a 96-well plate for 24 h at 37°C and 5% CO_2_. Subsequently, the cells were treated with TiO_2_ NTs at concentrations ranging from 100 μg/ml to 600 μg/ml (TiO_2_-equivalent dose). After 24 h, cytocompatibility was quantitatively assessed by CCK-8 assay (Dojindo, Japan) using a microplate reader (Thermo Scientific Multiskan GO). Cell viability was evaluated based on the following equation: cell viability (%) = OD_test_/OD_control_×100, where OD_test_ represents the absorbance of the treatment and OD_control_ represents the absorbance of the control. For evaluation of the cytotoxicity of DOX-loaded TiO_2_ NTs of different sizes, the cells were treated with DOX-loaded TiO_2_ NTs at concentrations ranging from 0 to 17 μg/ml (DOX-equivalent dose). Viability was again evaluated by measuring the absorbance at 450 nm using a microplate reader.

### 2.6 Evaluation of intracellular uptake of DOX-loaded TiO_2_ NTs of different sizes and FITC-loaded TiO_2_ NTs of different sizes by *in vitro* cell imaging and flow cytometry

SKOV-3 cells were used to investigate the potential intracellular uptake of TiO_2_ NTs of different sizes. The cells were seeded onto six-well plates and cultured in RPMI 1640 medium supplemented with 10% fetal bovine serum for 24 h. Subsequently, the medium was removed from each well, and 2 ml aliquots of DOX-loaded TiO_2_ NTs or FITC-loaded TiO_2_ NTs were added to the culture medium at the IC50 dose and left for 3 h. After the cells had been washed with PBS three times, the cell nuclei were stained with DAPI for 30 min, followed by washing with PBS three times. Fluorescence images of the DOX-loaded and FITC-loaded TiO_2_ NTs of different sizes were recorded by inverted fluorescence microscopy (Olympus, BH2-RFCA, Japan). To quantitatively examine cellular uptake in SKOV-3 cells, the cells were seeded onto six-well plates and cultured in RPMI 1640 medium containing 10% fetal bovine serum for 24 h. Subsequently, the medium was removed from each well, and 2 ml aliquots of DOX-loaded TiO_2_ NTs or FITC-loaded TiO_2_ NTs were added to the culture medium at the IC50 dose and left for 3 h, followed by washing with PBS three times. The cells were collected and analyzed by flow cytometry (Beckman Coulter Gallios).

## 3 Results and discussion

### 3.1 Preparation and characterization of TiO_2_ NTs and DOX-loaded TiO_2_ NTs of different sizes

**FIGURE 1:**
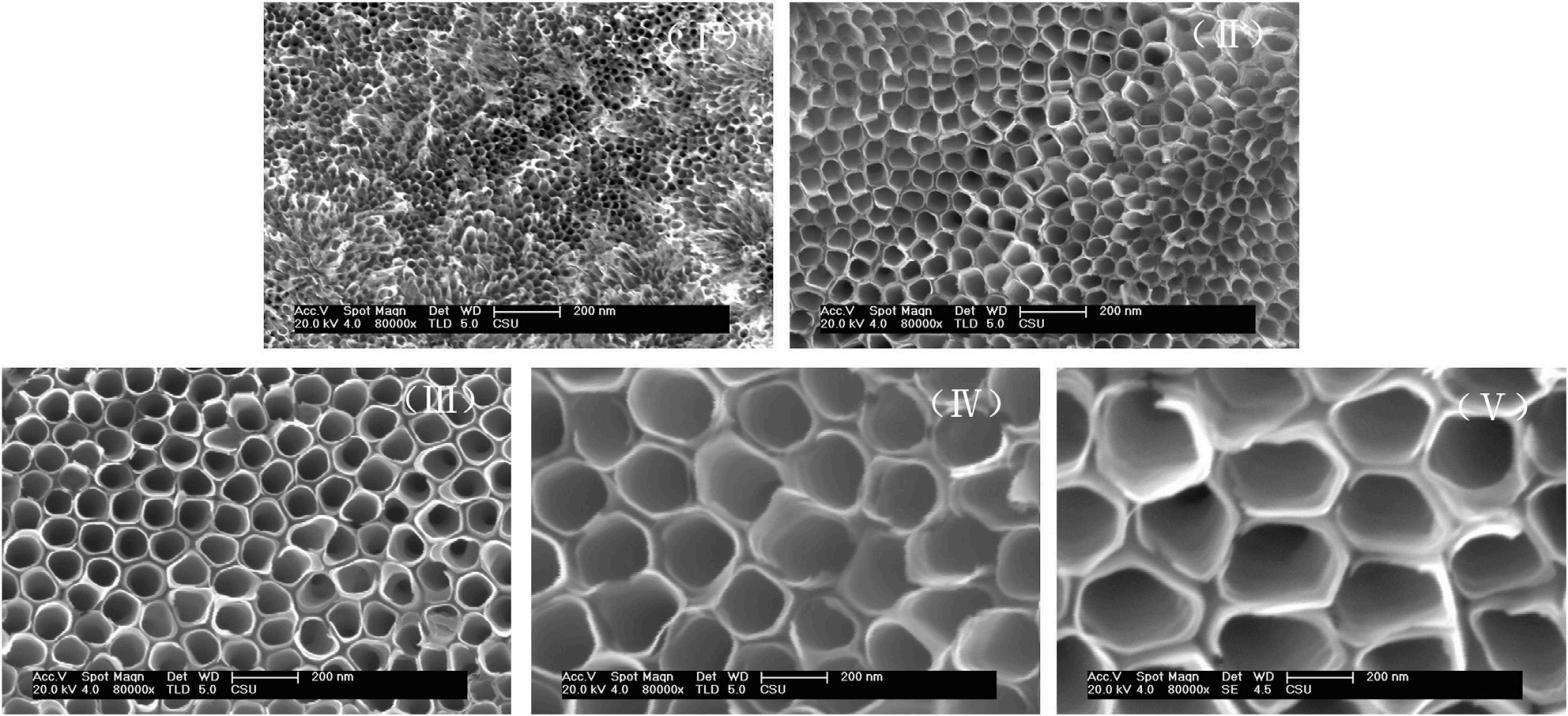
Representative SEM images of TiO_2_ NTs prepared at different voltages. TiO_2_ NTs anodized with 0.5 wt% NH_4_F, 10 vol% H_2_O, and 0.9 vol% H_2_SO_4_ in ethylene glycol at 50 V (I), 70 V (II), 100 V (III), 120 V (IV), and 150 V (V).

Highly structurally ordered TiO_2_ NTs were prepared by the anodization method as described in our previous work (Wang et al., 2014). The formation of the TiO_2_ NTs was driven by the electrolyte composition and voltage. In particular, F^−^ and the selected voltages were employed to improve hole mobility. The morphology of TiO_2_ NTs under application of different anodization voltages was confirmed by SEM. Figure 1 shows SEM images of the prepared TiO_2_ NTs of different sizes. TiO_2_ NTs of approximately 25 nm (I), 50 nm (II), 75 nm (III), 100 nm (IV), and 200 nm (V) in diameter were obtained by varying the anodization voltage. The layer thickness of the TiO_2_ NTs remained almost constant as their diameter increased. Compared with the formation of TiO_2_ NTs with a diameter of 25 nm (I) at 50 V, the diameter of the TiO_2_ NTs reached 200 nm (V) as the voltage was increased to 150 V. Higher voltages were found to accelerate the increase in diameter. The results revealed that the diameter of the TiO_2_ NTs was controlled by the anodization voltage when other conditions were held constant. In previous work by others, bundles of TiO_2_ NTs with diameters in the range of 170–220 nm have also been obtained using a similar method, which indicates the stability and reliability of the preparation method (Shirazi-Fard et al., 2021). The morphology of the NTs was not prone to collapsing under different voltages due to the optimized anodizing conditions. TiO_2_ NTs with good morphology were prepared successfully with a short reaction time (within 10 min), which is conducive to the efficient preparation of TiO_2_ NTs. The high-volume capacity of TiO_2_ NTs might provide exciting opportunities to maximize drug-loading capacity in order to control drug release and improve the anticancer effect. Importantly, with the benefits of the excellent performance of this approach to controlled growth, TiO_2_ NTs could produce greatly improved antitumor effects *in vitro*.

**FIGURE 2:**
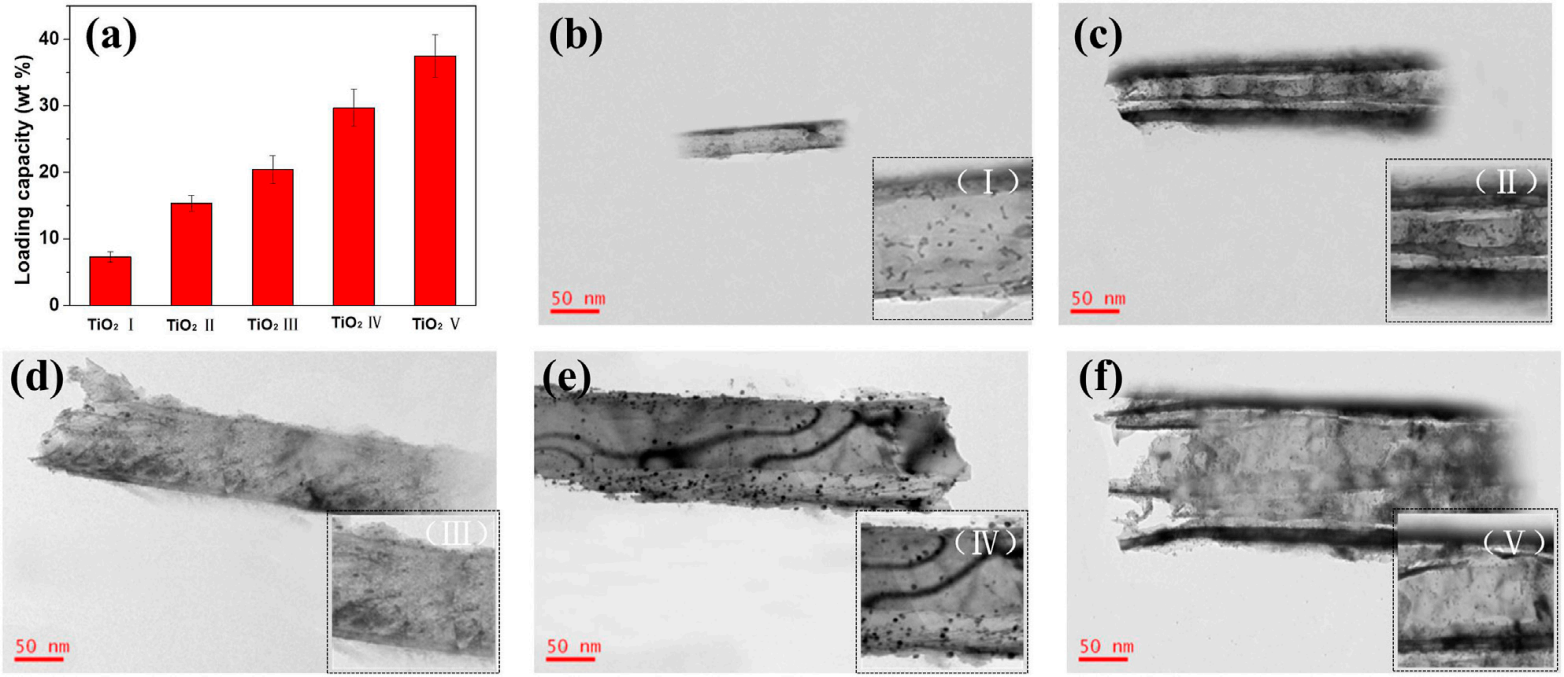
**(A)** Comparison of the DOX-loading capacity of TiO_2_ NTs of different sizes (bars represent mean ± SD; n = 3). **(B–F)** Representative TEM images of DOX-loaded TiO_2_ NTs of different sizes: **(B)** I, **(C)** II, **(D)** III, **(E)** IV, and **(F)** (V) were anodized at 50 V, 70 V, 100 V, 120 V, and 150 V, respectively. Bottom-right insets display local amplification of DOX-loaded TiO_2_ NTs.

The DOX-loading behavior of TiO_2_ NTs of different sizes after anodization and annealing was investigated through TEM analysis for elucidation of the DOX-loading process. Consistent with the SEM results, the DOX-loaded TiO_2_ NTs showed the same NT morphology undeor TEM (Figure 2). The TiO_2_ NTs were individual small NTs after ultrasonic treatment and filtration. There were abundant tiny black dots coating the walls of the TiO_2_ NTs, which were attributed to the loaded DOX. These black dots were observed to be well distributed throughout the support surface of the walls of the TiO_2_ NTs, with a uniform distribution of particle size. The formation of these black dots was a result of the hydrophobic agglomeration of DOX. The morphology of the NTs remained robust, without any deterioration even upon calcination and ultrasound. The loading capacity of TiO_2_ NTs of different sizes was determined by ultraviolet–visible spectroscopy. Figure 2A shows the DOX-loading capacity of TiO_2_ NTs of different sizes. The smallest TiO_2_ NTs (I) exhibited DOX-loading efficiency of 7.3 wt%, while the largest (V) provided excellent capacity of up to 37.5 wt%. Between these sizes, DOX-loading capacity was calculated to be 15.3 ± 1.2 μg/ml for TiO_2_ NTs of size II, 20.4 ± 2.1 μg/ml for size III, and 29.7 ± 2.8 for size IV. DOX could easily be introduced into the NTs via molecular diffusion. The loading efficiency of the largest TiO_2_ NTs (V) was five times greater than that of the smallest (I). In contrast, saturated loading capacity has been attained by previously reported carbon nanotubes, with a capacity of 83 μg/mg of DOX−TiO_2_ NTs having been obtained in the case of previously reported TiO_2_ NTs. In this work, a similar drug-loading capacity to that of other TiO_2_ NTs was observed, which indicates that the TiO_2_ NTs prepared as described here possess efficient and stable loading capacity (Kafshgari et al., 2019). An increase in their size might contribute to an increase in DOX-loading capacity. The results showed that TiO_2_ NTs of larger sizes accommodated high-capacity loads much more successfully and that the structure of the NTs constitutes a powerful tool for drug loading.

**FIGURE 3:**
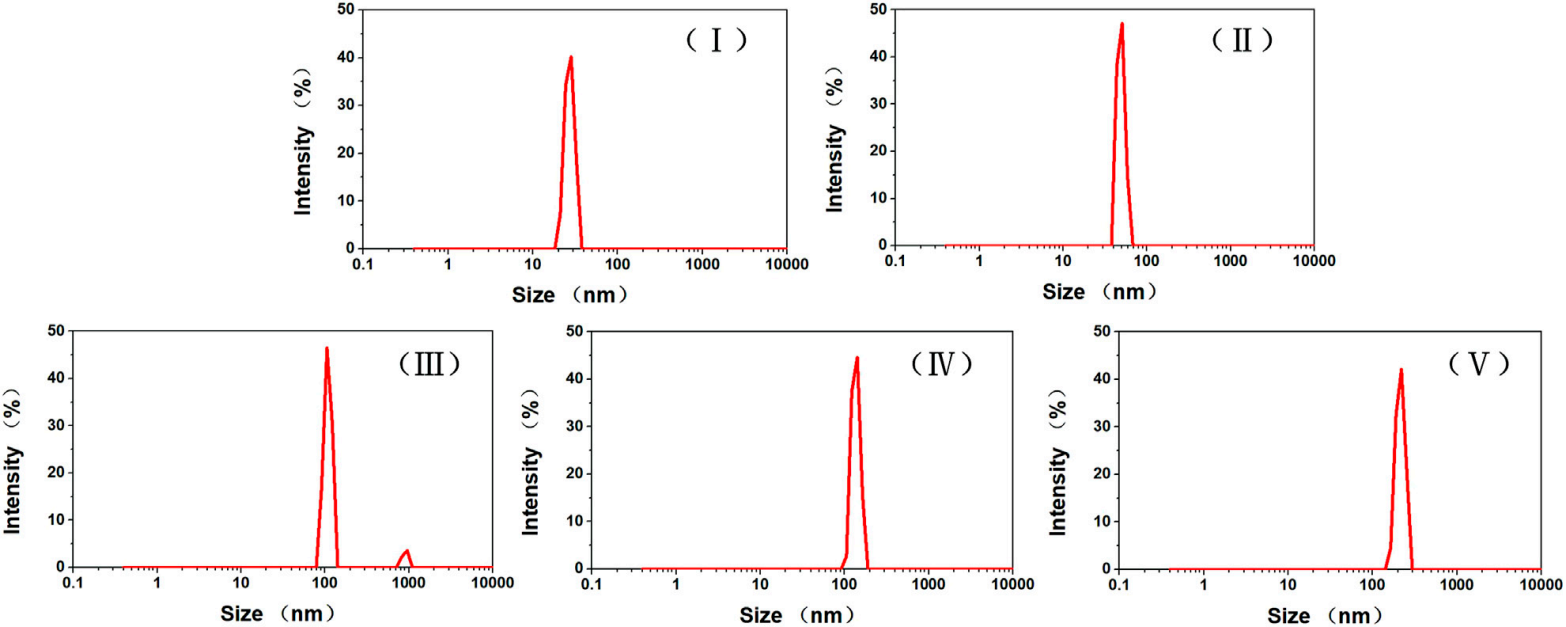
Size distributions of TiO_2_ NTs of different sizes in PBS (pH 7.4). TiO_2_ NTs of sizes I, II, III, IV, and V were anodized at 50 V, 70 V, 100 V, 120 V, and 150 V, respectively.

Further confirmation of the sizes of DOX-loaded TiO_2_ NTs in aqueous solution was obtained by carrying out DLS measurement, and the stability of TiO_2_ NTs of different sizes under aqueous conditions was also assessed (Figure 3). PDI values of 0.435, 0.373, 0.562, 0.453, and 0.304 were ascribed to TiO_2_ NTs of sizes I, II, III, IV, and V, respectively. The PDI values established by DLS were relatively small, which indicated the narrow size distribution. Due to the aggregation of TiO_2_ NTs under hydration state, the measured sizes of these TiO_2_ NTs were clearly larger than their actual sizes. It was found that the sizes measured by DLS were larger than the sizes measured by TEM in all cases. However, the pattern was similar, in that the TiO_2_ NTs prepared at high voltages were larger than those prepared at low voltages. Thus, the results were in good concordance with the previously obtained data. The particle size distribution was relatively narrow, and the TiO_2_ NTs showed good dispersion stability in aqueous medium. Therefore, ultrasonic treatment was considered to be an effective way to disperse the TiO_2_ NTs. In addition, ultrasonic treatment was convenient; more importantly, it was environmentally friendly; and it did not lead to possible structural damage. This was thought to be attributable to the tunable anodization parameters and the controlled growth of the NTs. The hollow structure was capable of providing sufficient hydrophobic space for DOX loading.

### 3.2 Drug release behavior of DOX-loaded TiO_2_ NTs of different sizes

**FIGURE 4:**
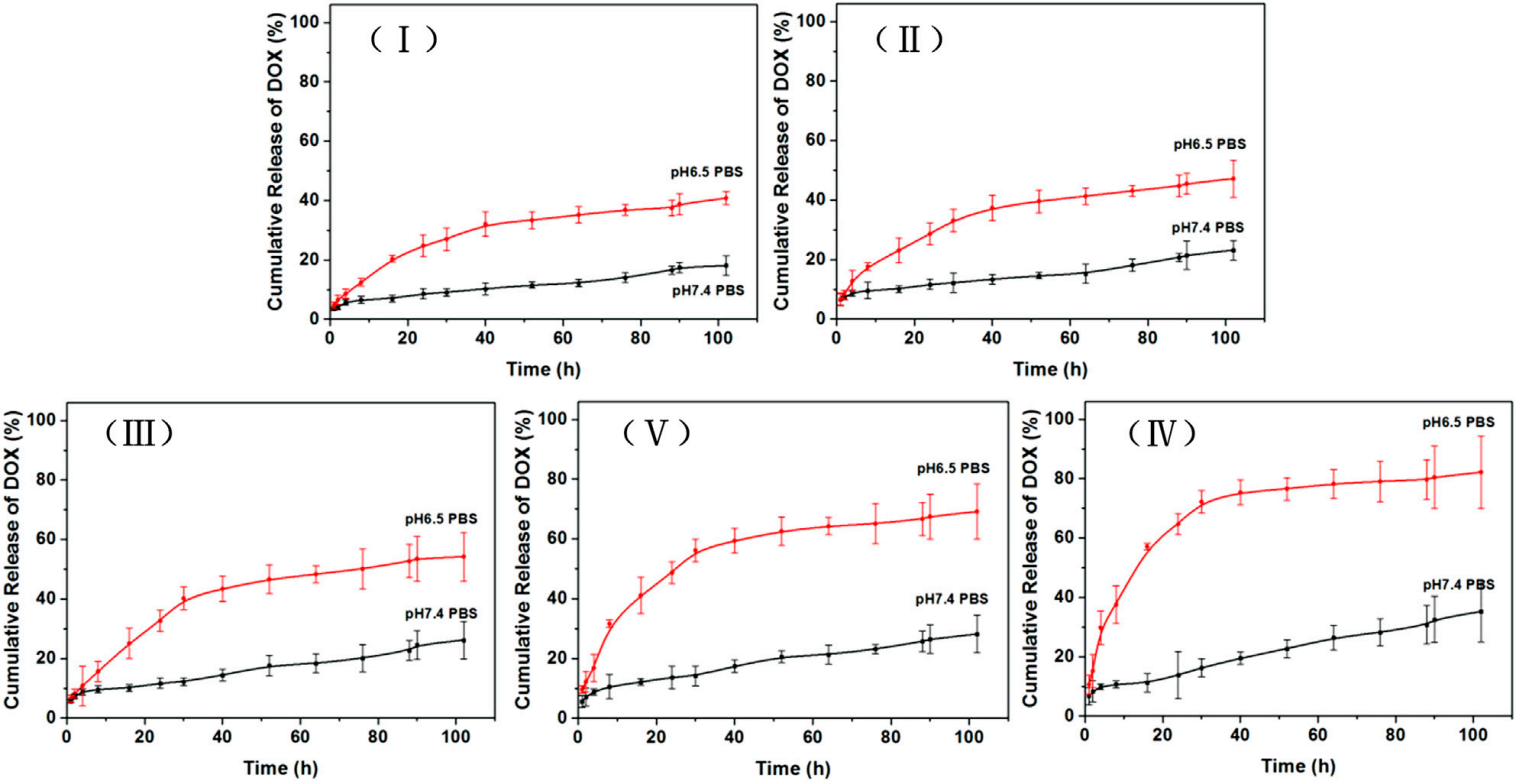
Cumulative percent release of DOX over time in PBS of pH 6.5 (mimicking the pH of the cancer tissue microenvironment) and PBS of pH 7.4 (mimicking the pH of physiological plasma). Data plotted are means ± SD (*n* = 3). TiO_2_ NTs of sizes I, II, III, IV, and V were anodized at 50 V, 70 V, 100 V, 120 V, and 150 V, respectively.

The cumulative DOX release rates of TiO_2_ NTs of the five different sizes are shown in Figure 4. The different DOX-loaded TiO_2_ NTs exhibited different *in vitro* release rate characteristics in PBS at pH 6.5 and 7.4. Overall, it can be seen that the lower pH value led to a higher cumulative percentage release of DOX. The smallest TiO_2_ NTs (I) exhibited significantly slower DOX release from the NT cavity. Specifically, only 40% of the drug was released into the acidic medium after 100 h. Therefore, the small DOX-loaded TiO_2_ NTs possessed a sustained and extended release profile. In the case of the largest TiO_2_ NTs (V), 80% of the DOX was released after 100 h at pH 6.5, but only 20% of the DOX was released after 100 h at pH 7.4. This clear increase in the amount of DOX released might be attributed to the large size of the TiO_2_ NTs, which favored subsequent drug diffusion and the rapid release of the drug from the NT cavity. In addition, the higher cumulative percentage release of DOX at the lower pH might be attributed to the higher solubility of DOX in an acidic environment. For carbon nanotube drug carriers, 90.66% of the total amount of DOX has been reported to be released over 24 h in an acidic microenvironment (Karthika et al., 2018). In other reported cases of TiO_2_ NTs, 96% of the DOX has been found to be released after 24 h (Shirazi-Fard, Mohammadpour, Zolghadr, and Klein, 2021). It seems that the TiO_2_ NTs prepared as described here exhibit a slow release rate compared to other reported NTs. However, these results could be interpreted in consideration of the finding that TiO_2_ NTs of larger size are endowed with stable drug-loading ability and controllable release of DOX in an acidic medium.

### 3.3 Biocompatibility of TiO_2_ NTs

**FIGURE 5:**
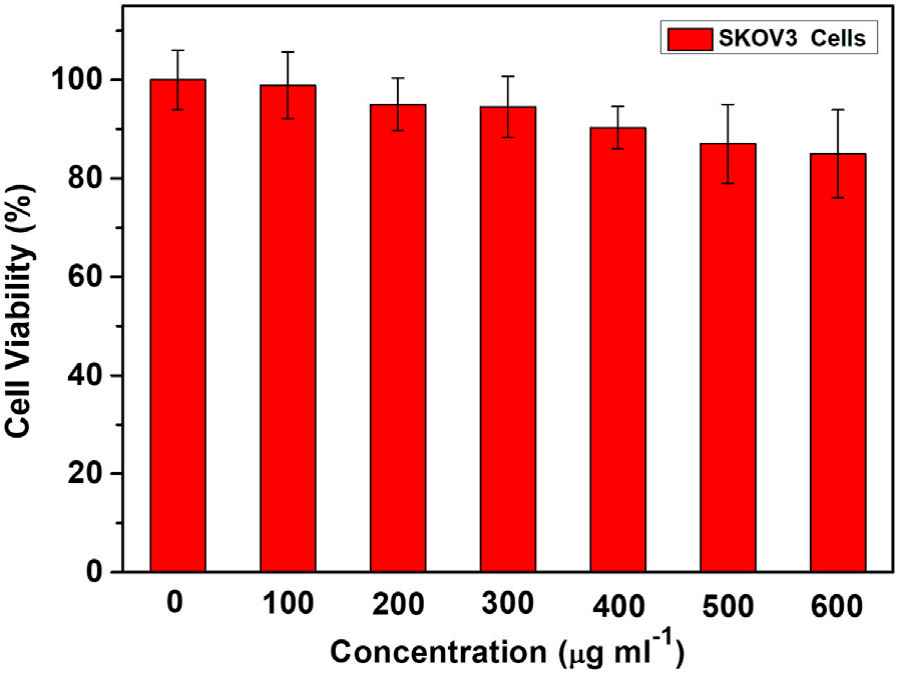
Cell viability with doses of unloaded TiO_2_ NTs for SKOV-3 cells, measured by CCK-8 assay. Each bar represents the mean ± SD (*n* = 6).

The cytocompatibility of unloaded TiO_2_ NTs was investigated to explore the potential cytotoxic effect. Based on the results of CCK-8 assay (Figure 5), cytotoxicity was not detectable at low doses. Furthermore, a high level of cell viability was observed (>90%) even at doses of up to 600 μg/ml. Therefore, the TiO_2_ NTs are biocompatible and do not induce any cytotoxic effect by themselves. These findings provide evidence that this approach is expected to provide an effective and safe drug delivery platform.

### 3.4 Investigations of cytotoxicity and intracellular drug release for DOX-loaded TiO_2_ NTs of different sizes

**FIGURE 6:**
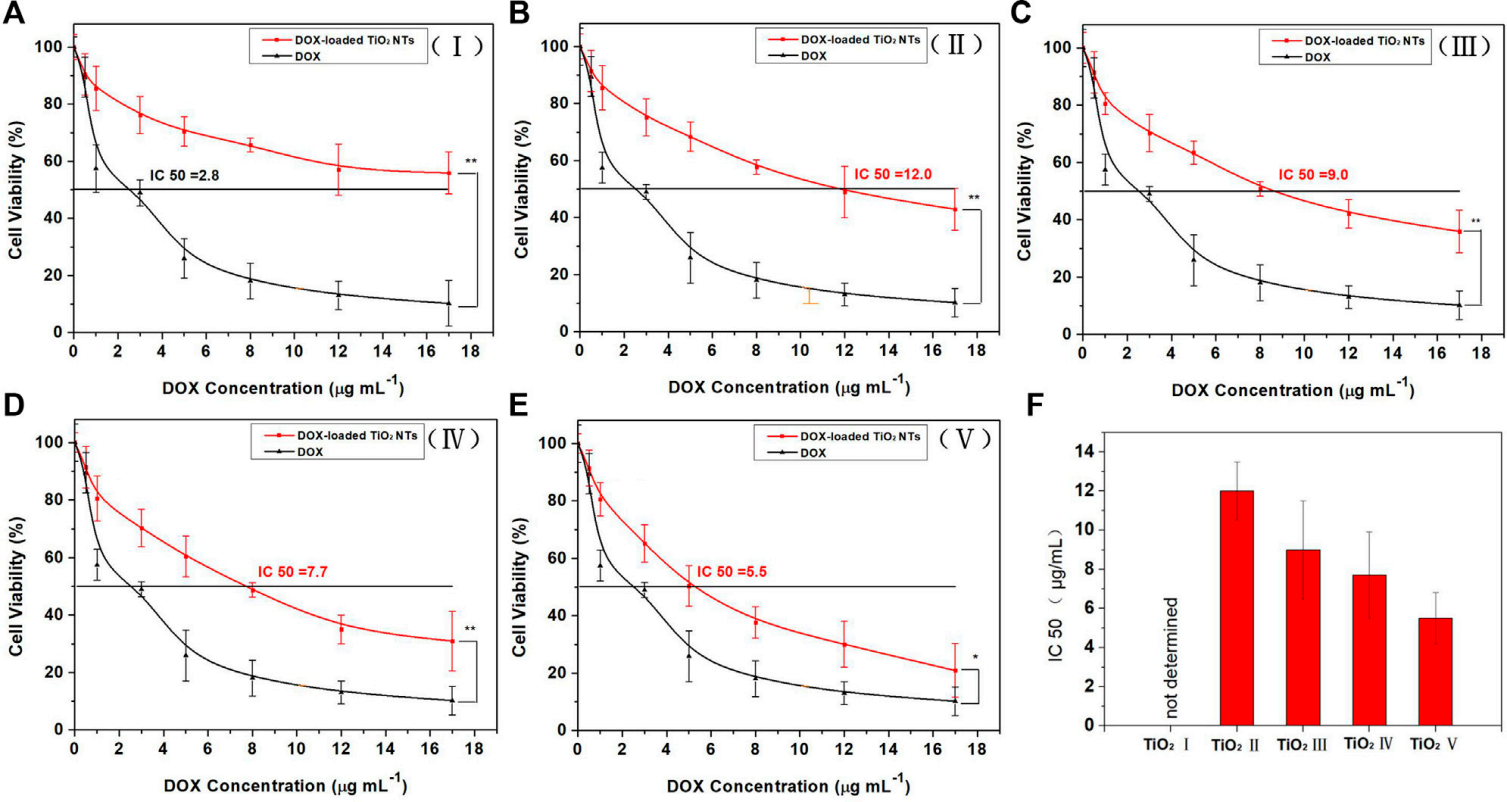
Cytotoxicity evaluation with SKOV-3 cells for DOX-loaded TiO_2_ NTs of sizes **(A)** I, **(B)** II, **(C)** III, **(D)** IV, and **(E)** V. **(F)** IC50 values of DOX-loaded TiO_2_ NTs (I, II, III, IV, and V). The cells were treated over 12 h with DOX or DOX-loaded TiO_2_ NTs. *p < 0.05; **p < 0.001 (in comparison with free DOX). The DOX treatment dose was based on DOX weight. Each bar represents the mean ± SD (*n* = 6). TiO_2_ NTs of sizes I, II, III, IV, and V were anodized at 50 V, 70 V, 100 V, 120 V, and 150 V, respectively.

The dose-dependent results of the cell viability test are shown in Figure 6. At the same DOX concentrations, DOX-loaded TiO_2_ NTs were associated with higher rates of cell viability than free DOX. The reason might be that cells treated with free DOX underwent rapid inhibition of cell growth, resulting in lower rates of cell viability. DOX-loaded TiO_2_ NTs were found to be less efficient than free DOX in terms of cell growth inhibition, most likely due to poor uptake by the cells and the lag in the release of DOX from the NTs. Free DOX could be transported into cells via passive diffusion, while DOX-loaded TiO_2_ NTs were probably taken up via the endocytosis pathway, which is time-consuming. The difference between free DOX and DOX-loaded TiO_2_ NTs in terms of cell viability rates was statistically significant after 12 h of incubation at concentrations ranging from 0 to 17 ug/ml. This experiment also demonstrated that the TiO_2_ NTs released DOX in a sustained manner, rather than as a single burst. Different IC50 values were obtained after treatment with DOX compared to DOX-loaded TiO_2_ NTs of different sizes. When incubated with SKOV-3 cells *in vitro*, large DOX-loaded TiO_2_ NTs were more effective than small NTs in interfering with cell growth, and the IC50 value decreased to 5.5 μg/ml (Figure 6). IC50 values were calculated to be approximately 12.0 μg/ml for TiO_2_ NTs of size II, 9.0 for size III, and 7.7 for size IV. Use of the smallest DOX-loaded TiO_2_ NTs delayed DOX release, and the IC50 value was not determined within the tested concentration range. Thus, higher concentrations of DOX in larger TiO_2_ NTs can easily be synthesized for optimal antitumor activity. For this purpose, the cell viability rate was concordant with the drug release behaviors of DOX-loaded TiO_2_ NTs of different sizes. The gradually increase in anticancer activity of DOX-loaded TiO_2_ NTs implies that the NT structure provides the advantages of sustained and controlled drug release and high biocompatibility, which can overcome the limitations of DOX relating to its high toxicity and poor water solubility.

**FIGURE 7:**
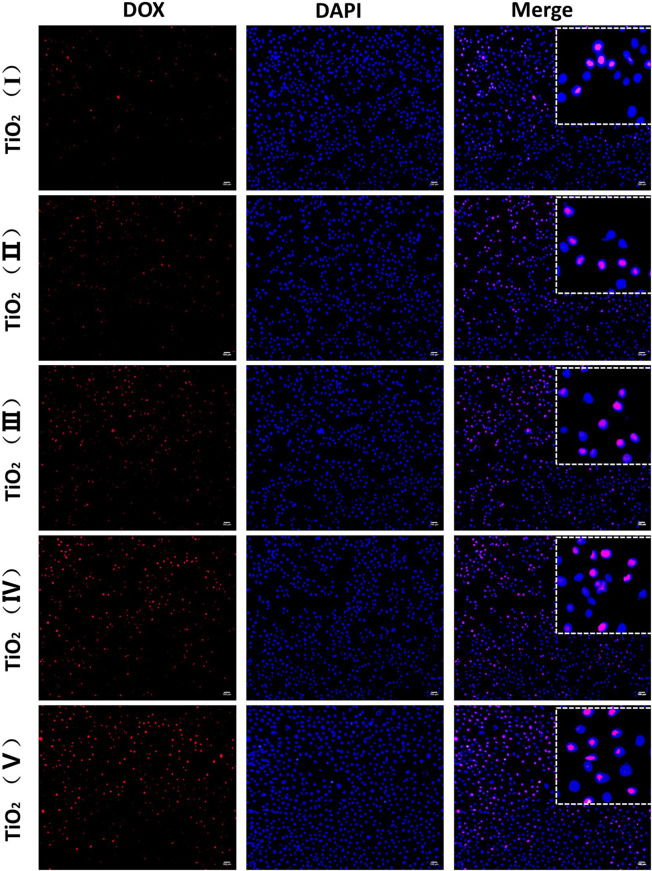
Fluorescence microscopy imaging of SKOV-3 cells treated with DOX-loaded TiO_2_ NTs of different sizes for 3 h. DOX-loaded TiO_2_ NTs appear in red, and the nuclei were stained with DAPI blue. Scale bar: 100 μm. TiO_2_ NTs of sizes I, II, III, IV, and V were anodized at 50 V, 70 V, 100 V, 120 V, and 150 V, respectively. DOX: *λ*_ex_ = 485 nm and *λ*_em_ = 585 nm; DAPI: *λ*_ex_ = 340 nm and *λ*_em_ = 485 nm.

DOX mainly works by entering the nucleus and intercalating with DNA for clinical prevention of DNA replication. Therefore, the amount of uptake of DOX from TiO_2_ NTs determines their ability to kill cancer cells and represents the delivery capacity of the TiO_2_ NTs. To verify that TiO_2_ NTs of different sizes could effectively deliver and release DOX, intracellular DOX distribution was analyzed by fluorescence imaging. As shown in Figure 7, DOX was mainly located in the nucleus. Almost all of the DOX fluorescence (red) overlapped with nucleus fluorescence (blue). When TiO_2_ NTs of different sizes were introduced into an acidic tumor environment, this acidic environment accelerated the release of DOX from the NTs. SKOV-3 cells incubated with DOX-loaded TiO_2_ NTs underwent different changes in fluorescence intensity for NTs of different sizes. Specifically, higher fluorescence was observed in the case of SKOV-3 cells incubated with larger TiO_2_ NTs, which shows that the larger TiO_2_ NTs released DX into the cells at a higher rate into the cells. Therefore, the results prove that the larger TiO_2_ NTs provided a rapid drug supply, a finding which was in good agreement with previous results on the drug release behavior of TiO_2_ NTs. Compared to the larger TiO_2_ NTs, the fluorescence intensities were reduced when the cells were incubated with DOX-loaded TiO_2_ NTs of small sizes, which could be explained by the limited drug release rate of the small NTs, due to the slow diffusion and release of the drug from the NT cavity.

**FIGURE 8:**
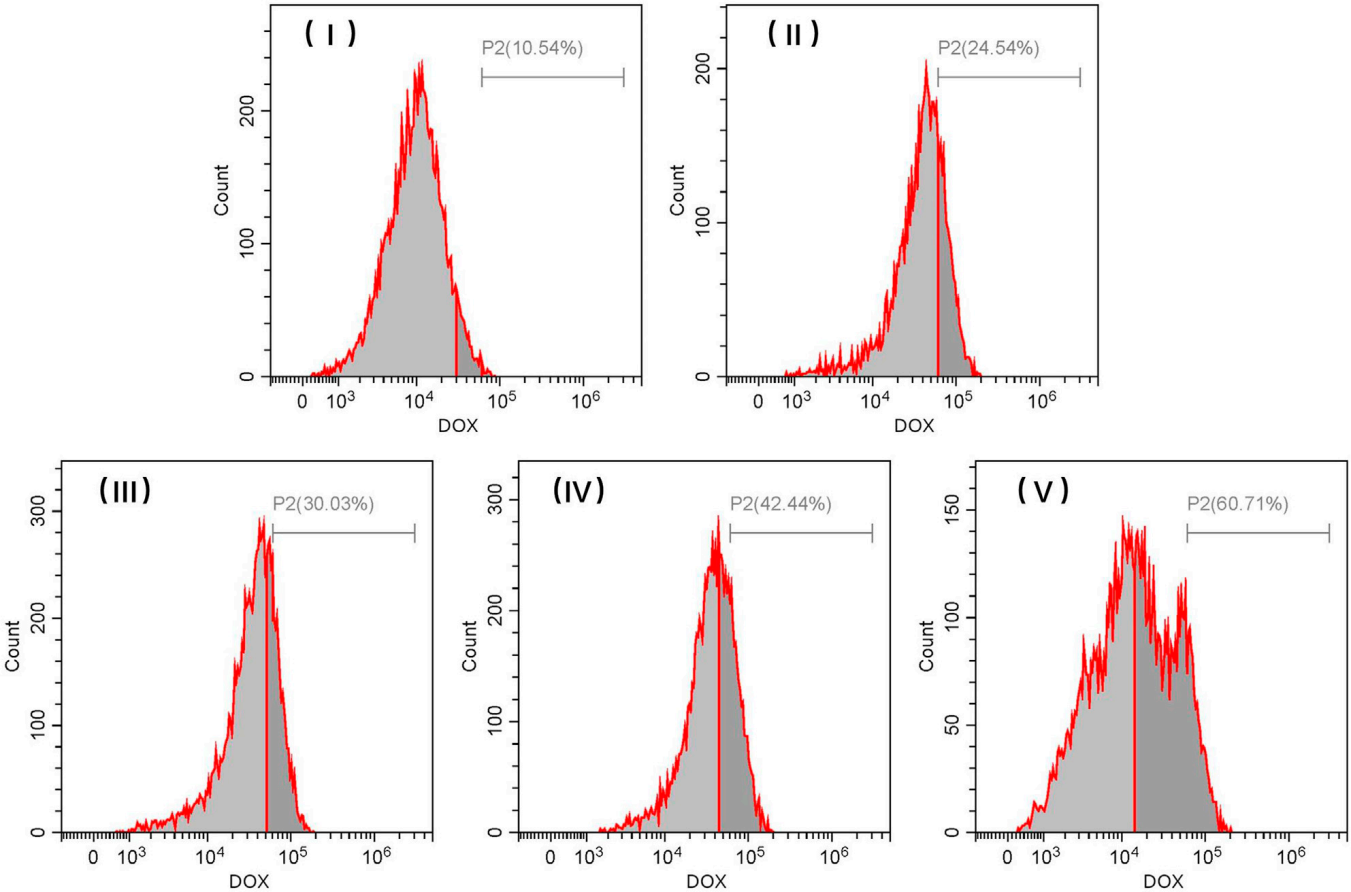
Representative flow cytometric histograms for SKOV-3 cells after incubation with different sizes of DOX-loaded TiO_2_ NTs for 3 h. TiO_2_ NTs of size I, II, III, IV, and V were anodized at 50 V, 70 V, 100 V, 120 V, and 150 V, respectively.

Flow cytometry analysis further confirmed the different rates of internalization associated with DOX-loaded TiO_2_ NTs of different sizes, which were illustrated by the notable increase in DOX fluorescence. Although fluorescence imaging for DOX-loaded TiO_2_ NTs indicated the variation in intracellular uptake for DOX-loaded TiO_2_ NTs of different sizes, these values were not quantified. As shown in Figure 8, intracellular DOX fluorescence intensity was much stronger in cells treated with large TiO_2_ NTs than in cells treated with the small TiO_2_ NTs. The flow cytometry results indicated that TiO_2_ NTs of size V produced a fluorescence intensity 5.7-, 2.5-, 2.0-, and 1.4-fold higher than those of size I, II, III, and IV, respectively. Importantly, these results suggest that the intracellular uptake of DOX-loaded TiO_2_ NTs was size-dependent. Based on the open tube structure and excellent biocompatibility of TiO_2_ NTs, it was further determined that the large TiO_2_ NTs could be a promising candidate for drug loading and sustained release in combination with anticancer therapy.

### 3.5 Intracellular drug release for FITC-loaded TiO_2_ NTs of different sizes

**FIGURE 9:**
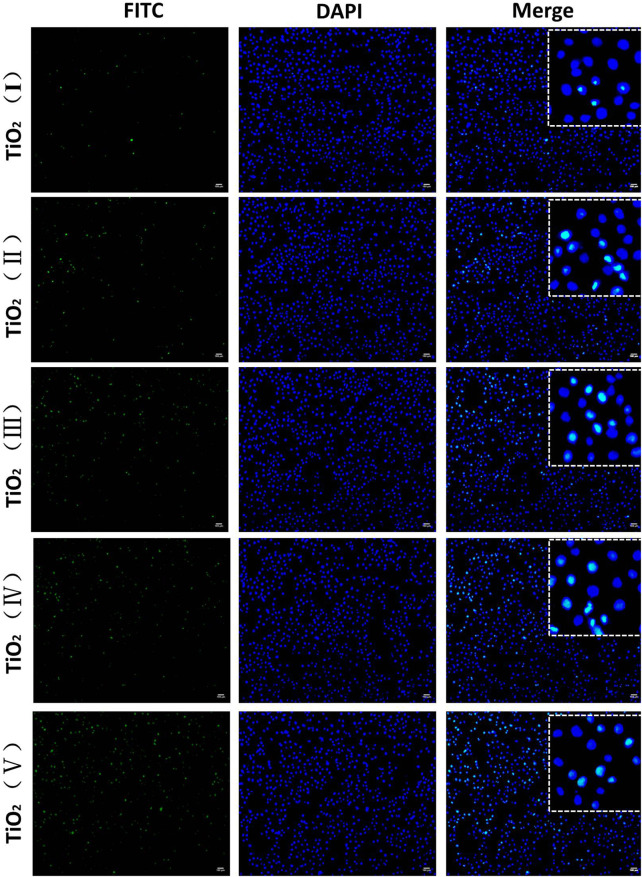
Fluorescence microscopy imaging of SKOV-3 cells treated with FITC-loaded TiO_2_ NTs of different sizes for 3 h. FITC-loaded TiO_2_ NTs appear in green, and the nuclei were stained with DAPI blue. Scale bar: 100 μm. TiO_2_ NTs of size I, II, III, IV, and V were anodized at 50 V, 70 V, 100 V, 120 V, and 150 V, respectively. FITC: *λ*_ex_ = 488 nm and *λ*_em_ = 525 nm; DAPI: *λ*_ex_ = 340 nm and *λ*_em_ = 485 nm.

*In vitro* uptake studies were further performed by incubating SKOV-3 cells with FITC-loaded TiO_2_ NTs of different sizes. As shown in Figure 9, green fluorescence produced by FITC-loaded TiO_2_ NTs of different sizes was observed within SKOV-3 cells. FITC-loaded TiO_2_ NTs of different sizes exhibited approximately similar trends in the results of fluorescence imaging to DOX-loaded TiO_2_ NTs. As expected, the results indicated that small TiO_2_ NTs contributed to lower rates of cellular internalization of FITC than large TiO_2_ NTs, and that TiO_2_ NTs of size V had a stronger effect than other TiO_2_ NTs on cellular entry. Fluorescence microscopy and flow cytometry tests indicated the successful uptake of NTs by SKOV-3 cells, with size-dependent regulation.

**FIGURE 10:**
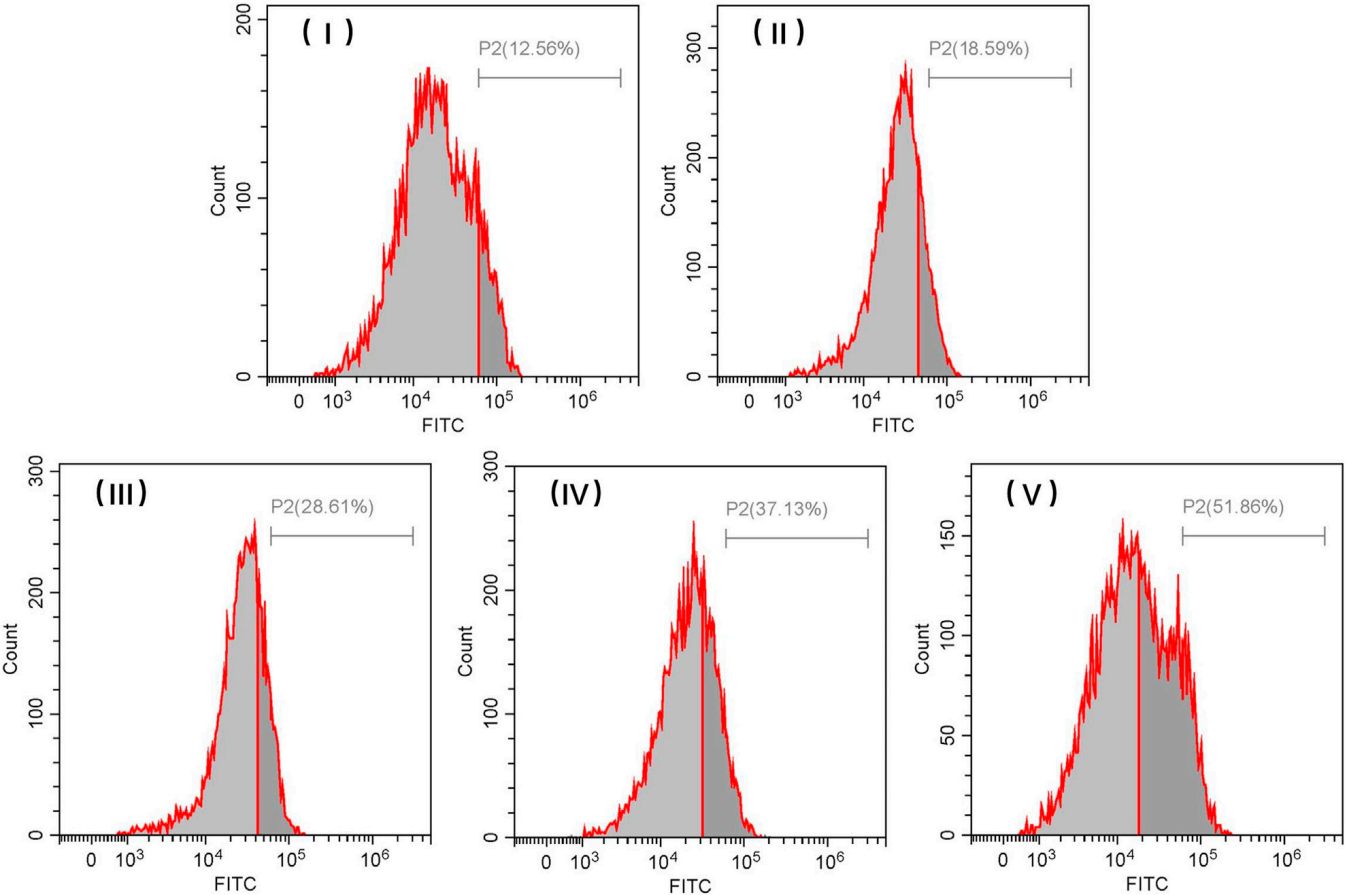
Representative flow cytometric histograms of SKOV-3 cells after incubation with FITC-loaded TiO_2_ NTs of different sizes for 3 h. TiO_2_ NTs of size I, II, III, IV, and V were anodized at 50 V, 70 V, 100 V, 120 V, and 150 V, respectively.

The cellular uptake rates associated with FITC-loaded TiO_2_ NTs of different sizes with a 3-h treatment were also quantified by flow cytometry using the fluorescent feature of FITC (Figure 10). The cellular uptake rates of FITC-loaded TiO_2_ NTs of different sizes were found to be similar to those indicated by the flow cytometry analysis of DOX-loaded TiO_2_ NTs of different sizes. These positive findings prove that this system could act as a high-efficiency drug delivery system in cancer therapy. Taken together, the data indicate that larger TiO_2_ NTs are more likely to release the drugs than their smaller counterparts and that the drug-loading capacity of NTs is clearly related to their size. Beyond demonstrating the value of large TiO_2_ NTs as highly efficient drug load-bearers, the methods developed in this study also allow for control over the size of TiO_2_ NTs by varying the anodizing voltage, enabling precise regulation of drug loading and release.

## 4 Conclusion

In summary, we have designed and successfully prepared TiO_2_ NTs of different sizes by the anodization method. The tubular structure of TiO_2_ NTs makes for stable drug loading, and the larger TiO_2_ NTs exhibited higher drug-loading efficiency. *In vitro* release assays of DOX-loaded TiO_2_ NTs of different sizes under simulated physiological and acidic conditions indicated that TiO_2_ NTs were pH-responsive. The effectiveness of DOX-loaded TiO_2_ NTs of different sizes in terms of cytotoxicity was proven by CCK-8 measurements, in which the most efficient cell-killing capacity was ascribed to the larger TiO_2_ NTs. We assume that the larger TiO_2_ NTs can store a larger quantity of drug molecules and allow rapid and immediate release of these after being triggered by the presence of acidic conditions. Fluorescence imaging also indicated the superior intracellular uptake ability of drug-loaded TiO_2_ NTs of larger size. Flow cytometry analysis further demonstrated the superior cellular uptake associated with larger DOX-loaded TiO_2_ NTs. Our results reveal the details of drug loading and release, as well as differences in intracellular uptake behaviors between tumor cells and between NTs of different sizes; this provides new insight into the optimal design of tubular structures as drug carriers for drug-loading in the treatment of tumors.

## Data Availability

The original contributions presented in the study are included in the article/Supplementary Material; further inquiries can be directed to the corresponding author.
